# Intrachannel anterograde snare traction combined with a clip-and-line system for polyps in the appendix cavity

**DOI:** 10.1055/a-2602-3288

**Published:** 2025-06-18

**Authors:** Zhiying Gao, Zhenguo Pan, Jian Zhao, Feng Pan

**Affiliations:** 1 91596 Department of Gastroenterology, The Affiliated Huaiʼan No.1 Peopleʼs Hospital of Nanjing Medical University, Huaiʼan, China; 2 91596 Department of Doppler Ultrasound, The Affiliated Huai’an No.1 People’s Hospital of Nanjing Medical University, Huaiʼan, China

A-57-year-old man with no symptoms underwent a colonoscopy due to a history of colon polyp. The procedure revealed a 1.5-cm polypoid eminence with an indistinct boundary to the base in the appendix cavity. Abdominal computed tomography (CT) showed a normal appendix. After obtaining informed consent, endoscopic removal of the appendix lesion was performed ( Video 1 ).

An improved operation approach involved a clip-and-line system and a snare device pierced by floss in the instrument channel which was used to excise the ileocecal lesion.Video 1

First, a single clip-and-line traction system by the instrument channel was applied to the lesion, for adequate exposure of the submucosal layer ( Fig. 1 , Fig. 2 ). Second, after the submucosal injection, a snare device was used to excise entirely the ileocecal lesion, which was pierced by floss in the instrument channel ( Fig. 3 ). Finally, the lesion was extracted from the intestinal lumen using a snare device ( Fig. 4 ). The procedure was successfully performed within 10 minutes.

**Fig. 1:**
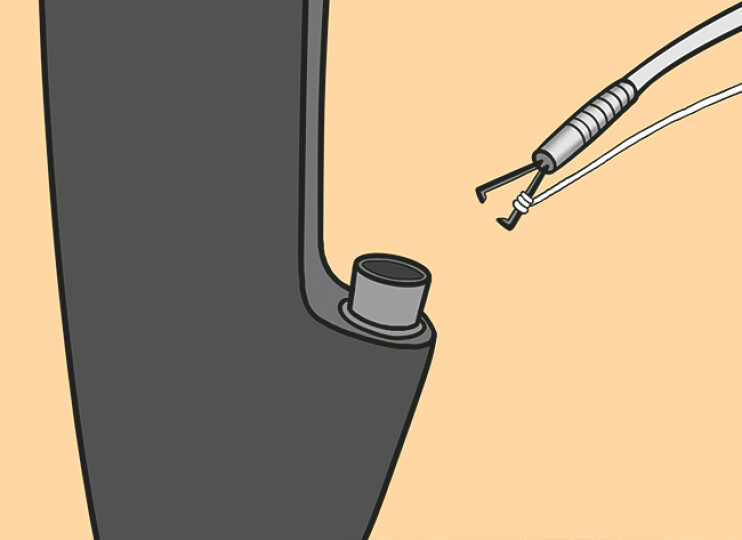
A single clip-and-line system through the instrument channel approaches the lesion.

**Fig. 2:**
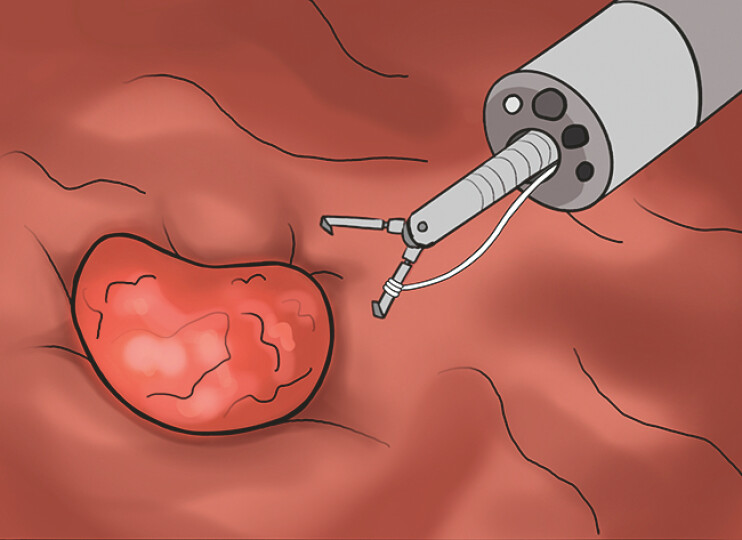
The ileocecal lesion was exposed adequately by the single clip-and-line system.

**Fig. 3:**
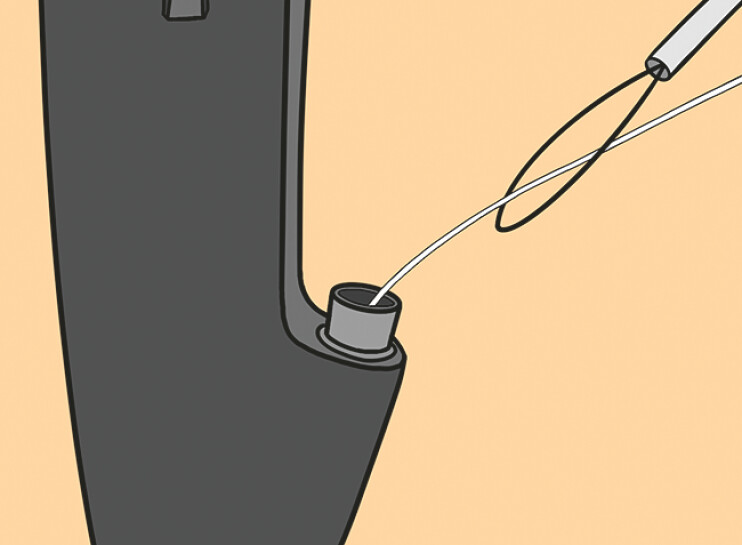
A snare device was pierced by floss through the instrument channel.

**Fig. 4:**
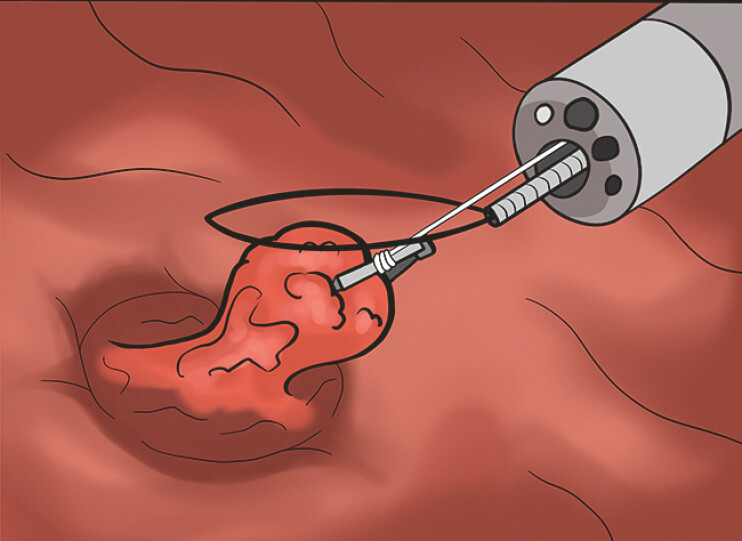
Anterograde snare traction combined with a clip-and-line system for polyps in the appendix cavity excised entirely the ileocecal lesion.

Postoperative histopathological analysis confirmed the presence of sessile serrated lesions (SSLs) in the ileocecal lesion. The patient made a swift recovery and was discharged 5 days after the procedure.

When confined entirely within the appendiceal lumen, these lesions are nearly undetectable by conventional colonoscopy. When the lesion is completely confined to the appendix, removal of the polyp is often performed along with removal of the appendix 1 . Our team performed a super minimally invasive procedure to remove the lesion completely while preserving the appendix that considered as an important immune organ. This case highlights the security and effectiveness of a super minimally invasive procedure to the appendix lesion when such lesions are identified in the colon.

Endoscopy_UCTN_Code_TTT_1AQ_2AD_3AB

## Notes

Endoscopy E-Videos https://eref.thieme.de/e-videos
*E-Videos* is an open access online section of the journal *Endoscopy* , reporting on interesting cases and new techniques in gastroenterological endoscopy. All papers include a high-quality video and are published with a Creative Commons CC-BY license. Endoscopy E-Videos qualify for HINARI discounts and waivers and eligibility is automatically checked during the submission process. We grant 100% waivers to articles whose corresponding authors are based in Group A countries and 50% waivers to those who are based in Group B countries as classified by Research4Life (see: https://www.research4life.org/access/eligibility/ ). This section has its own submission website at https://mc.manuscriptcentral.com/e-videos .

## References

[1] Yao J Liu K Zhao G Endoscopic management of multiple sessile serrated lesions in both the ileocecal region and the appendix cavity Endoscopy 2024 56 E841 E842 39357840 10.1055/a-2418-0499 PMC11446631

